# *MDM2* polymorphism associated with the development of cervical lesions in women infected with Human papillomavirus and using of oral contraceptives

**DOI:** 10.1186/1750-9378-9-24

**Published:** 2014-07-18

**Authors:** Carolina MM Amaral, Katerina Cetkovská, Ana PAD Gurgel, Marcus V Cardoso, Bárbara S Chagas, Sérgio SL Paiva Júnior, Rita de Cássia Pereira de Lima, Jacinto C Silva-Neto, Luiz AF Silva, Maria TC Muniz, Valdir Q Balbino, Antonio C Freitas

**Affiliations:** 1 Laboratory of Molecular Studies and Experimental Therapy, Department of Genetics, Universidade Federal de Pernambuco, Pernambuco, Brazil; 2 Department of Biology, Faculty of Medicine, Masaryk University, Brno, Czech Republic; 3 Laboratory of Human Molecular Genetics, Department of Genetics, Universidade Federal de Pernambuco, Pernambuco, Brazil; 4 Molecular and Cytological Research Laboratory, Department of Histology, Universidade Federal de Pernambuco, Pernambuco, Brazil; 5 Institute of Biological Sciences and Health, Federal University of Alagoas, Alagoas, Brazil; 6 Laboratory of Molecular Biology of Center of Pediatric Oncohaematological, University of Pernambuco, Pernambuco, Brazil

**Keywords:** *MDM2*, HPV, Oral contraceptives, Cervical cancer

## Abstract

**Background:**

The *MDM2* gene is the major negative regulator of p53, a tumor suppressor protein. Single nucleotide polymorphism in promoter region of *MDM2* gene leads to increased expression resulting in higher levels of MDM2 protein. This event increases the attenuation of the p53 pathway. Polymorphisms in this gene can interfere in the regulation of cellular proliferation. We evaluated whether *MDM2* SNP309 (rs2278744) associated or not with the use of oral contraceptive can heighten susceptibility to development of cervical lesions in women HPV infected.

**Methods:**

*MDM2* SNP309 (rs2278744) was genotyped in a total of 287 patients using the PCR-RFLP technique. The results were analyzed by UNPHASED v.3.121 and SNPStats programs.

**Results:**

The three groups (SIL, LSIL and HSIL) showed no significant differences in either genotype or allelic frequencies for *MDM2* polymorphisms, except when HSIL was compared with LSIL (p = 0.037; OR = 1.81). Furthermore, in the analysis of contraceptives, a significant association was found between the use of contraceptives and the *MDM2* variant in the development of high-grade cervical lesions for the TG genotype (p = 0.019; OR = 2.21) when HSIL was compared with control. When HSIL was compared with LSIL (p = 0.006; OR = 2.27).

**Conclusion:**

The results of this study suggest that *MDM2* SNP309 might be a good marker for assessing the progression of LSIL to HSIL. In addition, they also show that oral contraceptives alone, did not have any effect on the progression or development of cervical lesions. However, they may act synergistically with *MDM2* SNP309 (rs2278744) and HPV infection in the development of cervical lesions.

## Background

Cervical cancer is the third most frequent cancer and the second most common cause of death among women worldwide [1]. Its prevalence is especially high in developing countries, which account for 86% of all cases [1]. In Brazil, there are more than 17,500 new cases every year and 4,812 deaths [2]. Currently, the main screening method for cervical cancer is the Papanicolau (Pap) smear test which is based on an analysis of cervical cell morphology. The cervical cancer precursor is the squamous intraepithelial lesion (SIL) that may be either a high squamous intraepithelial lesion (HSIL) or a low squamous intraepithelial lesion (LSIL) [3].

It is well established that the major etiological factor in cervical cancer is Human papillomavirus (HPV) infection [4,5]. HPV DNA is detected in over 95% of the cases of invasive cervical cancer worldwide [6]; however, the type of HPV, as well as genetic and environmental factors, can influence the persistence of HPV infection and the progression of lesions to cervical cancer [7-9]. The use of oral contraceptives has been put forward as a cofactor in the development of cervical cancer and in making the user predisposed to the disease [9]. It is believed that steroids hormones interact with a specific DNA sequences on transcriptional regulatory sites on the HPV DNA increasing or suppressing the transcription of various genes [10]. Estradiol and progesterone can be responsible for modulation the host genes that are involved in immune response to HPV16 [11]. Moreover, the association with cervical cancer depends the type-specific HPV infection [12]. The effects of oral contraceptive on cervical cancer are transient because the association increases with duration of use [13,14] and decreases after discontinuation [11,13,14].

The HPV E6 oncoprotein that is involved in cervical cancer makes use of the cellular ubiquitin–protein ligase E6-AP to target the p53 transcription factor for degradation [15]. The human homolog of mouse double minute 2 (mdm2) protein is the main negative regulator of the p53 function [16]. Mdm2 targets the p53 protein by ubiquitination for its degradation and suppresses p53 by inhibiting its trans-activation function [17]. The level of p53 is regulated through an auto-regulatory feedback loop where p53 induces the transcription of *MDM2*.

The *MDM2* regulation of disorders is associated with the development of cancer, which is confirmed by the evidence that *MDM2* was over-expressed in some human cancers [18,19]. *MDM2* single nucleotide polymorphism SNP (*rs2279744*) has already been described as being associated with several types of cancer [20,21], and also with accelerated tumorigenesis and poor prognosis [22]. This polymorphism is located in position 309 in the first intron of the *MDM2* oncogene, which serves as a transcriptional enhancer region. When T is replaced with G, this increases the affinity of the Sp1 transcription factor and it has been shown that cells carrying the T309G genotype have a 2-3-fold increase in *MDM2* mRNA and protein synthesis. This leads to the abrogation of p53 tumor suppressor activity and the development of cancer [23].

Our study sought to determine whether the presence of polymorphism in the *MDM2* gene associated or not with the use of oral contraceptives can heighten susceptibility to the development of cervical lesions in women HPV infected.

## Results

This study was conducted with 212 cases of cervical lesions from North-East Brazil who underwent cervical cancer screening and 75 individuals that does not have lesions acting as a control group.

The age of the patients included in the current study ranged from 15–77 the average age being 33 and the mean was at 34.02 + 11.05 years. In accordance with their HPV types, 78% of all the patients were infected with high-risk HPV against 22% that were infected with low-risk. In the control group, 65% were infected with high-risk HPV and 35% with low-risk HPV. In the LSIL group, 74% were infected with high-risk HPV and 26% with low-risk HPV. In the HSIL group, 98% were infected with high-risk HPV and 2% with low-risk.

The SNP were evaluated in compliance with the Hardy-Weinberg principle. The frequencies of each allele and genotype are given in Table 1.

**Table 1:** ***MDM2 *** **(rs2278744) polymorphism in patients with HPV and cervical lesions of low degree (LSIL) and high degree (HSIL) and healthy patients**

	**Cases**			**Normal cytology **	***p*-value; OR [95% C.I.]**			
	**Total SIL**^**a**^	**LSIL**^**b**^	**HSIL**^**c**^	**Control**^**d**^	**Total SIL vs. C**	**LSIL vs. C**	**HSIL vs. C**	**HSIL vs. LSIL**
*SNP309 rs2278744*								
Alelles								
T	265 (0.62)	165 (0.63)	100 (0.61)	95 (0.63)	Reference	Reference	Reference	Reference
G	156 (0.38)	95 (0.37)	61 (0.39)	55 (0.37)	0.80; 1.04 [0.71-1.54]	0.97; 0.99 [0.65-1.50]	0.67; 1.11 [0.70-1.75]	0.55; 1.14 [0.74-1.75]
Genotypes								
TT	80 (0.37)	54 (0.42)	26 (0.30)	30 (0.40)	Reference	Reference	Reference	Reference
TG	105 (0.51)	57 (0.44)	48 (0.61)	35 (0.47)	0.83; 0.85 [0.48-1.49]	0.92; 1.11 [0.60-2.04]	0.33; 1.58 [0.80-3.13]	*0.11; 1.75 [0.95-3.20]
GG	27 (0.12)	19 (0.15)	8 (0.09)	10 (0.13)	0.83; 0.99 [0.43-2.28]	0.92; 0.95 [0.39-2.30]	0.33; 0.92 [0.32-2.69]	0.33; 0.92 [0.32-2.69]

*p* < 0.05 – statistically significant.

^a^SIL: Squamous intraepithelial lesions.

^b^LSIL: Low-grade squamous intraepithelial lesions.

^c^HSIL: High-grade squamous intraepithelial lesions.

^d^C: Control - Normal cytology.

*In overdominant model the risk to TG genotype was 1.81 (1.03-3.16, 95% CI).

Patients with both LSIL and HSIL were included in the SIL group. The three groups (SIL, LSIL and HSIL) showed no significant differences in either genotype or allelic frequencies for *MDM2* SNP309 polymorphism, except when HSIL was compared with LSIL for the *MDM2* gene. The difference was significant (p = 0.037; OR = 1.81, 95% CI 1.03-3.16) with regard to the TG genotype when TT was used as a reference-point. This showed that the significant genetic model was over-dominant, with the heterozygote being at a greater risk of evolution of LSIL for HSIL than either of the homozygotes. Although this is a no intuitive model, there is a precedent for other genes acting in this manner (see Discussion).

An examination was carried out to establish the possible influence of the use of hormonal contraceptives on the susceptibility of women to the development of cervical lesions (Table 2). There was no association between the use of contraceptives and LSIL and HSIL.

**Table 2:** Distribution of use of contraceptive according to the SIL, LSIL, HSIL and controls

	**Cases**			**Normal cytology **	***p*-value; OR [95% C.I.]**			
	**Total SIL**^**a**^	**LSIL**^**b**^	**HSIL**^**c**^	**Control**^**d**^	**Total SIL vs. C**	**LSIL vs. C**	**HSIL vs. C**	**HSIL vs. LSIL**
***Variable***								
Use of contraceptive								
No	167 (0.68)	109 (0.65)	58 (0.75)	55 (0.68)	OR = 0.99 [0.55-1.79]	OR = 1.14 [0.62-2.13]	OR = 0.69 [0.32-1.48]	OR = 0.60 [0.31-1.14]
Yes	78 (0.32)	95 (0.35)	19 (0.25)	25 (0.32)	*p* = 1.0	*p* = 0.66	*p* = 0.37	*p* = 0.10

*p* < 0.05 – statistically significant.

^a^SIL: Squamous intraepithelial lesions.

^b^LSIL: Low-grade squamous intraepithelial lesions.

^c^HSIL: High-grade squamous intraepithelial lesions.

^d^C: Control - Normal cytology.

Another examination was carried out to establish the effects of the use of hormonal contraceptives (together with variants of the *MDM2* gene) on the development of cervical lesions (Table 3). When the TT-GG genotypes were used as a reference-point, a significant difference was found between HSIL and the control groups [*p* < 0.05; OR = 2.21 (1.13-4.30)] and the TG genotype, for *MDM2* SNP309. A significant difference was also found in the TG genotype when HSIL was compared with LSIL. The risk ranged from 2.01 to 2.31 depending on the model used. For codominant model, the risk was 2.31; for dominant model, the risk was 2.01 and for overdominant model, the risk was 2.27 to TG genotype. Since the overdominant model was the most significant (*p* = 0.006). We also compared *MDM2* SNP309 allele distribution among oral contraceptive users and non-users between the three groups, LSIL, HSIL and normal cytology (Table 4).

**Table 3:** ***MDM2 *** **(rs2278744) polymorphisms in patients with HPV and cervical lesions of low degree (LSIL) and high degree (HSIL) and healthy patients with contraceptive as co-factor**

	**Cases**			**Normal cytology **	***p*-value; OR [95% C.I.]**			
	**Total SIL**^**a**^	**LSIL**^**b**^	**HSIL**^**c**^	**Control**^**d**^	**Total SIL vs. C**	**LSIL vs. C**	**HSIL vs. C**	**HSIL vs. LSIL**
*SNP309 rs2278744*								
Alelles								
T	254 (0.61)	165 (0.63)	89 (0.59)	92 (0.64)	Reference	Reference	Reference	Reference
G	158 (0.39)	95 (0.37)	63 (0.41)	52 (0.36)	0.81; 1.05 [0.71-1.54]	0.98; 0.99 [0.66-1.51]	0.68; 1.12 [0.70-1.75]	0.56; 1.15 [0.74-1.75]
Genotypes								
TT	74 (0.36)	54 (0.41)	20 (0.26)	30 (0.42)	Reference	Reference	Reference	Reference
TG	106 (0.51)	57 (0.44)	49 (0.64)	32 (0.44)	0.59; 0.74 [0.42-1.33]	0.99; 1.2 [0.55-1.91]	^1^0.06; 2.24 [1.08-4.63]	^2^0.02; 2.31 [1.21-4.41]
GG	26 (0.13)	19 (0.15)	7 (0.10)	10 (0.14)	0.59; 0.95 [0.41-2.21]	0.99; 0.98 [0.40-2.39]	0.06; 1.06 [0.34-3.25]	0.02; 1.07 [0.39-2.96]

p-value adjusted with contraceptive.

*p* < 0.05 – statistically significant.

^a^SIL: Squamous intraepithelial lesions.

^b^LSIL: Low-grade squamous intraepithelial lesions.

^c^HSIL: High-grade squamous intraepithelial lesions.

^d^C: Control - Normal cytology.

^1^In overdominant model the p-value was 0.019; OR = 2.21 (95% CI, 1.13-4.30).

^2^In dominant model the p-value was 0.02; OR = 2.01 (95% CI, 1.07-3.76) and in overdominant model, the p-value was 0.006; OR = 2.27 (95% CI, 1.25-4.10).

**Table 4:** **Comparison *MDM2 *** **SNP309 allele distribution among oral contraceptive users and non-users between LSIL, HSIL and normal cytology (control)**

	**Cases**									**Control**^**d**^		
***SNP 309 rs2278744***	**Total SIL**^**a**^			**LSIL**^**b**^			**HSIL**^**c**^					
Alleles	Yes	No	Total	Yes	No	Total	Yes	No	Total	Yes	No	Total
T	79 (0.58)	175 (0.63)	254 (0.61)	56 (0.57)	109 (0.68)	165 (0.63)	23 (0.60)	66 (0.58)	89 (0.59)	29 (0.60)	63 (0.66)	92 (0.64)
G	57 (0.42)	101 (0.37)	158 (0.39)	42 (0.43)	53 (0.32)	95 (0.37)	15 (0.40)	48 (0.42)	63 (0.41)	19 (0.40)	33 (0.34)	52 (0.36)
Genotypes												
TT	22 (0.32)	52 (0.38)	74 (0.36)	17 (0.35)	37 (0.46)	54 (0.41)	5 (0.26)	15 (0.27)	20 (0.26)	9 (0.37)	21 (0.44)	30 (0.42)
TG	35 (0.51)	71 (0.51)	106 (0.51)	22 (0.45)	35 (0.43)	57 (0.44)	13 (0.69)	36 (0.63)	49 (0.64)	11 (0.46)	21 (0.44)	32 (0.44)
GG	11 (0.17)	15 (0.11)	26 (0.13)	10 (0.20)	9 (0.11)	19 (0.15)	1 (0.05)	6 (0.10)	7 (0.10)	4 (0.17)	6 (0.12)	10 (0.14)

^a^SIL: Squamous intraepithelial lesions.

^b^LSIL: Low-grade squamous intraepithelial lesions.

^c^HSIL: High-grade squamous intraepithelial lesions.

^d^Control: Normal cytology.

## Discussion

In this study, we evaluated whether *MDM2* SNP309 (rs2278744) associated or not with the use of oral contraceptive can heighten susceptibility to development of cervical lesions.

The nature of the relationship between *MDM2* SNP309 (rs2278744) and cervical cancer is not clear. There have been few studies in this area and the conclusions are contradictory. We found that the differences in the frequency of *MDM2* SNP309 (rs2278744) allele and genotype were not significant between the LSIL and HSIL groups, when compared with the control group. This is consistent with the results of a single study with a Brazilian population that was carried out to test whether *MDM2* SNP309 (rs2278744) was associated with either the risk of cervical cancer or its diagnosis at an early age. In this study, Meissner et al. [24] compared 72 cervical carcinoma patients with 100 healthy patients and no association was observed between *MDM2* SNP309 (rs2278744) and cervical cancer. Our results lend weight to the conclusions of Meissner et al. [24] that *MDM2* SNP309 (rs2278744) may not be a risk factor for the development of cervical carcinogenesis.

However, our study found a statistically significant result of this SNP with the progression of LSIL to HSIL, which was not analyzed by Meissner et al. [24]. In contrast, recently, Singhal et al. [25] found association between higher frequency of G allele and cervical cancer in Indian women infected with HPV. In the study of a population in China, involving 167 cervical cancer and 223 controls, Jiang et al. [26] found an increased risk of cervical cancer associated with *MDM2* SNP309 (rs2278744) GG. Nunobiki et al. [27] investigated whether or not the combination of *MDM2* SNP309 (rs2278744) with HPV types could be associated with cervical lesions and cancer. Their study consisted of 102 LSIL, 41 HSIL and 52 normal (controls). *MDM2* SNP309 (rs2278744) was found to be associated with cervical carcinogenesis especially in the high-risk HPV group.

The divergence between the findings of these studies and the present study might be caused by racial, ethnic or samples differences in the populations studied, since in our study we worked with pre-cancerous lesions. According to a study carried out by Wo et al. [28], in the case of an Asian population, the mean frequency of 309G is 0.49 and 0.36 for the mixed population. It would be interesting to examine the reasons for the differences found in this study in greater depth.

When HSIL was compared with LSIL, the difference was significant and showed a risk of 1.81 with the TG genotype when the TT-GG genotype was used as a reference-point (overdominant model). The genetic model suggested by this data appears to be an over-dominant protective effect of *MDM2*. This model is also called ‘heterozygote disadvantage’, and although it may appear counterintuitive, a review suggests that this mode of action is perhaps more common than previously thought and cites numerous examples [29]. Indeed, the *MDM2* promoter polymorphism has been associated with the severity of HPV, and this also seems to follow a pattern of heterozygote disadvantage.

According to the *MDM2* polymorphism (SNP309) analysis, our study reported that there is a small discrepancy between control and LSIL groups. However, this difference is considerable between LSIL and HSIL groups. This suggest that *MDM2* SNP309 may be a good marker to assess the progression of LSIL to HSIL. More studies should be done to continue this hypothesis.

We also examined the role of contraceptives as an environmental co-factor in the risk of cervical lesions. Some studies did not observe any association between the use of oral contraceptives and cervical lesions [30-32]. However, several studies demonstrated that when combined with other factors the history of the use of contraceptives affects the development of cervical lesions, and the risk is increased with the duration of their use [33-36]. In our study, the use of contraceptives was either evaluated alone or together with *MDM2* SNP309 in women with HPV infection. No association was found when the use of contraceptives was evaluated alone. This finding was consistent with the results of Castle et al. [32], who did not find a higher risk of high-grade lesions or cervical cancer among 1160 women who used contraceptives compared with 2094 who did not use them. Hildesheim et al. [31] did not find oral contraceptives had an effect on cervical lesions when this co-factor was evaluated alone. Deacon et al. [30] observed that the use of oral contraceptives was not significantly associated with high-grade lesions. When comparing 211 HSIL and 1475 invasive cancer patients with 255 HPV- positive controls without lesions, Moreno et al. [35] found that those who had used contraceptives for less than 5 years did not demonstrate an increased risk of cervical cancer. Nevertheless, the risk increased 2.82- fold when the use was over a period of 5–9 years and 4.03- fold when the use lasted for 10 years or more. Brisson et al. [34] observed a relative 1.9 risk of high-grade lesions in patients that had used contraceptives for 6 years or more compared with those who never used them. Briton et al. [33], found that the risk was two-fold for users of 5 or more years. A study conducted by Luhn et al. [36] with 2783 women in various stages of cervical disease, showed that long-term oral contraceptive use (a period of more than 10 years) was associated with HSIL with the risk being 2.42- fold higher than those that had never used contraceptives.

These results are in contrast with the current study where the use of contraceptives alone was not associated with development of cervical lesions. Factors such as the discontinuous use of contraceptives and the duration of their use can explain this divergence. It is also possible that in studies where associations were found between the use of contraceptives and development of lesions and cervical cancer, there is also an interaction with the host genetic factors which were not analyzed.

When the use of contraceptives was examined with the presence of *MDM2* SNP309 in the development of cervical lesions, a risk of 2.21 was found in patients with HSIL and TG genotype when compared with control and *MDM2 MDM2* SNP309. When HSIL was compared with LSIL, the risk was 2.31 (codominant model), 2.01 (dominant model) and 2.27 (overdominant model). These results provide clues that contraceptives can act synergistically by increasing susceptibility to the development of high-grade cervical lesions. According to Bond and Levine [37], when the estrogen signaling pathway is activated in the presence of SNP309, the basal levels of the MDM2 in the cell are elevated, due to the affinity binding site of the transcription factor Sp1 created in the promoter region. As a result, the levels of p53 are reduced and the carriers of the G allele have a low cellular stress response. Thus, the activated estrogen signaling pathway is necessary for the G allele to accelerate the formation the tumor [37].

This is the first study that showed an association between the use of contraceptives and *MDM2* SNP309 (rs2278744) in the development of cervical lesions of women HPV infected. The findings corroborate the hypothesis that the use of contraceptives may be a co-factor together with HPV infection and *MDM2* polymorphism in the development of cervical lesions.

These findings reported that *MDM2* SNP309 (rs2278744) were significantly associated with women HPV infected that using oral contraceptive, suggesting that contraceptives act together with *MDM2* SNP309 (rs2278744) and HPV infection in the development of cervical lesions in women HPV infection. Additional studies should be conducted in order to prove that the polymorphism in *MDM2* gene may come to be used as a marker of the evolution of high grade lesions in women HPV infected.

## Methods

### Ethics statement

The study was approved by the "Research Ethics Committee of University of Pernambuco", Brazil, (HUOC/PROCAPE 64/2010) and was approved by the institutional review board of the University Hospital Oswaldo Cruz. All patients provided written informed consent prior to collection of sample. It was obtained informed written consent from the next of kin, caretakers, or guardians on behalf of the minors that were enrolled in this study.

### Studied subjects

The cervical smears were obtained from 287 patients from North-East Brazil who volunteered to take part in cervical cancer screening. All the samples included in this study were HPV positive. The control group consisted of 75 patients without cervical lesions and the case group consisted of 212 patients with cervical lesions which was divided into LSIL (130 - Low grade squamous intraepithelial lesions) and HSIL (82 - High grade squamous intraepithelial lesions). The patients signed a consent form that was approved by the ethics committee of the university (HUOC/PROCAPE 64/2010).

### Preparation of samples and DNA extraction

After being collected, the samples were placed in polyethylene tubes containing phosphate-buffered saline and stored at -20°C for further processing. DNA was extracted using the DNeasy Blood and Tissue Kit (Qiagen, São Paulo/Brazil) in accordance with the manufacturer's instructions.

### HPV detection

PCR was used for HPV DNA detection and involved the amplification of the viral L1 gene fragment using degenerated primers (MY09/MY11) [38,39]. The primer sequences were MY09- CGTCCMARRGGAWACTGATC and MY11- GCMCAGGGWCATAAYAATGG.

### MDM2 SNP *(*rs2279744) genotyping

**Figure 1:**
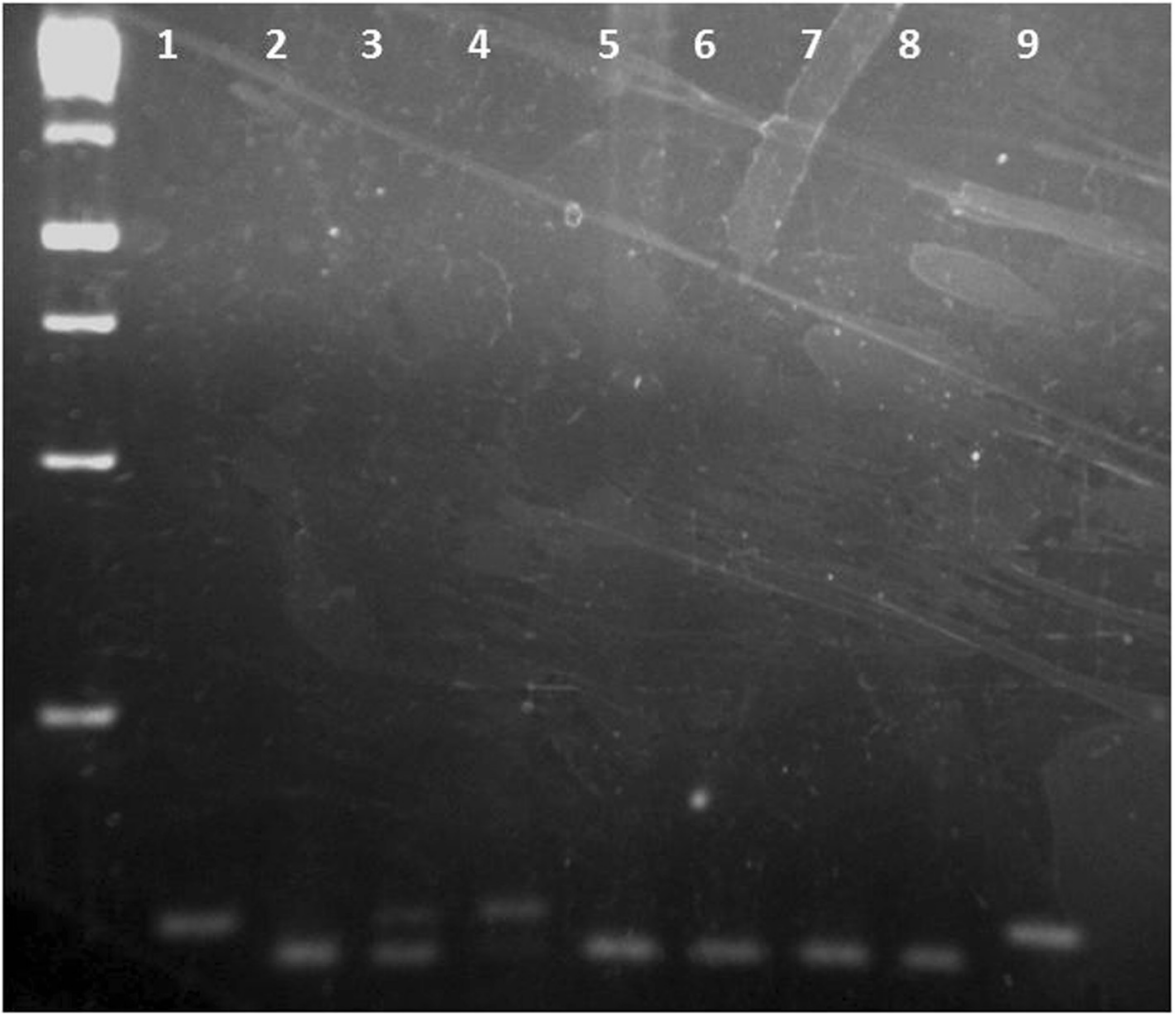
**Representative genotypes of *MDM2 *SNP (rs2278744).** *MDM2* SNP (rs2278744) - Wild-type homozygous TT produced a single 121 bp fragment (Columns 1,9), homozygous GG produced 2 fragments of 104 bp and 17 bp, which was not detected on the gel (Colunms 2,5,6,7,8) and heterozygot TG produced 3 fragments of 121 bp, 104 bp and 17 bp (Colunms 3,4).

**Figure 2:**
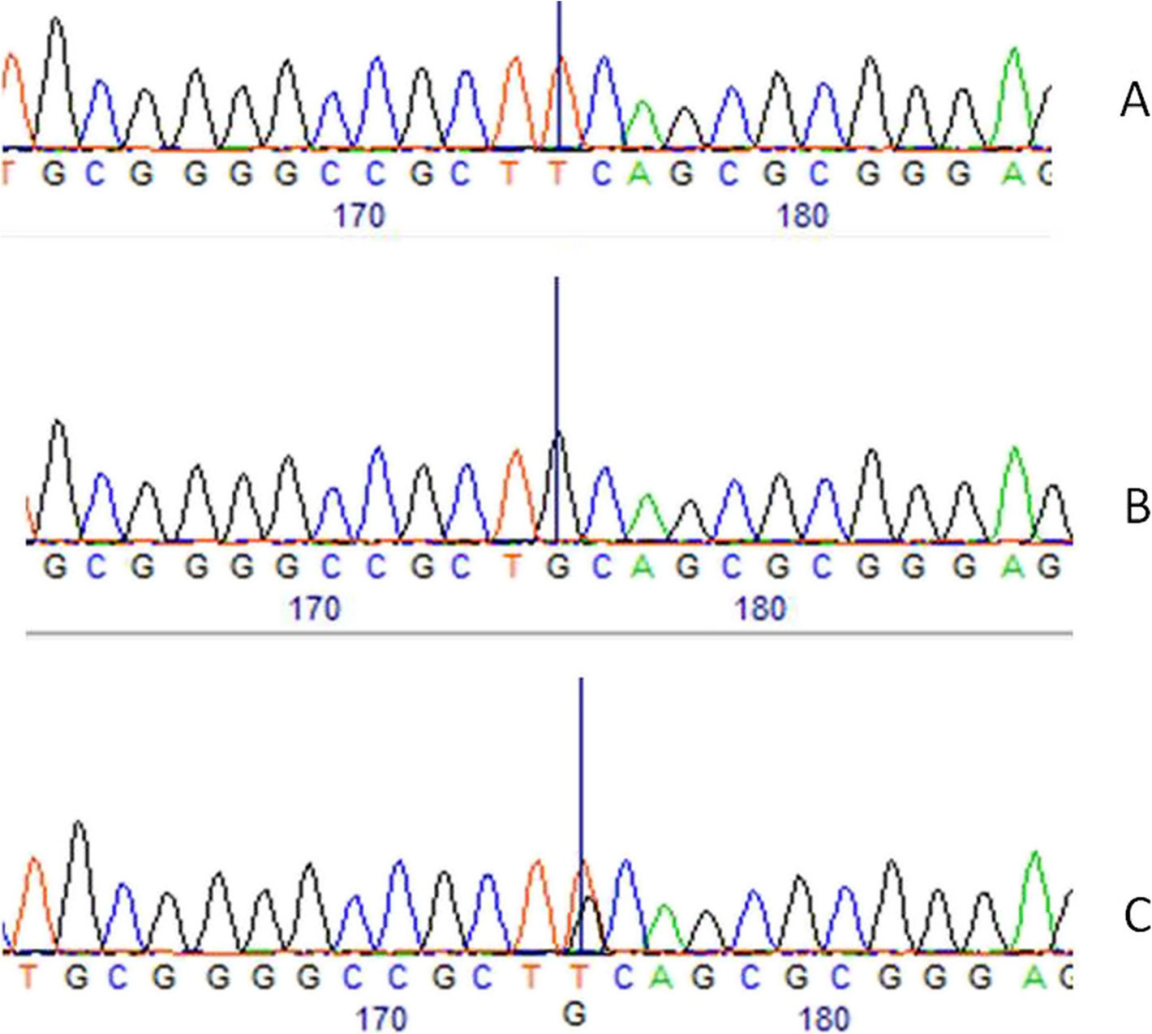
**Sequence analysis.** Comparisons of the three genotypes on the site of nucleotide change. **A** – Wild-type homozygous TT. **B** – Homozigous GG. **C** – Heterozygous TG.

A Primer- Introduced Restriction Analysis PCR (PIRA-PCR) assay was used for the genotyping of *MDM2* SNP309. This method is based on sense and antisense-primers, where there is a mismatch of the nucleotide close to a polymorphic site, which is introduced to create a restriction site. The antisense-primer introduced A to replace G at 2 bp from the polymorphic site to create a PstI restriction site. The primer sequences were 5’-GATTTCGGACGGCTCTCGCGGC-3’ (sense) and 5’-CATCCGGACCTCCCGCGCTG-3’ (antisense) [40]. 50 ng of the DNA template from each sample was amplified with 0.1 mM dNTP, 1X Taq reaction buffer, 1 mM MgCl2, 0.8 μM of each primer and 1U Taq DNA polymerase in a final volume of 25 μL. The amplification reaction was carried out under the following conditions: 95°C for 5 minutes for the initial denaturation of the genomic material, 35 cycles of 95°C for 30 seconds (denaturation), 66.5°C for 40 seconds (annealing), 72°C for 45 seconds (extension) for the amplification of the gene segment of interest and a cycle of 72°C for 10 minutes in the final extension of the fragment. The 121 bp PCR fragment was then digested for 16 hours by 5U PstI (Invitrogen, São Paulo/Brazil). The wild-type T allele produced a single 121 bp fragment and the polymorphic G allele produced two fragments of 104- and 17 bp. The product was analyzed on 2% agarose gel stained with ethidium bromide (Figure 1). The nucleotide change and the three genotypes can be visualized on sequences analysis that were shown on Figure 2.

### Statistical methods

The genetic frequency and association analysis between the comparison groups was performed with UNPHASED v.3.121 [41], whereas association analysis between these groups and risk factor (use of contraceptive) was conducted with SNPStats [42]. All the tests were two-tailed and the level of significance for all of the statistical results was set at p < 0.05.

### Ethical approval

This work has been approved by Ethics Committee on Human Research – Hospital Complex HUOC/PROCAPE (HUOC/PROCAPE 64/2010).

## Competing interest

The authors have no conflict of interest to declare.

## Authors’ contributions

The individual contribution of each author was performed as follows: CMMA performed all the experimental work and manuscript elaboration; KC, APADG, BSC and RCPL performed experimental support; MVC and SSLPJ performed the statistical analyzes; ACF, was responsible for orientation and coordination of this manuscript; MTCM; JCSN; LAFS and VQB, were the consultants of this manuscript. All authors read and approved the final manuscript.
